# Improvement of Spatial Memory Disorder and Hippocampal Damage by Exposure to Electromagnetic Fields in an Alzheimer’s Disease Rat Model

**DOI:** 10.1371/journal.pone.0126963

**Published:** 2015-05-15

**Authors:** Xiao Liu, Hongyan Zuo, Dewen Wang, Ruiyun Peng, Tao Song, Shuiming Wang, Xinping Xu, Yabing Gao, Yang Li, Shaoxia Wang, Lifeng Wang, Li Zhao

**Affiliations:** 1 Department of Experimental Pathology, Beijing Institute of Radiation Medicine, 27 Taiping Road, Haidian District, Beijing, China; 2 Beijing Key Laboratory of Bioelectromagnetism, Institute of Electrical Engineering, Chinese Academy of Sciences, 6 North Second Street, Zhongguancun, Beijing, China; Technion - Israel Institute of Technology, ISRAEL

## Abstract

Although some epidemiological investigations showed a potential association between long-term exposure of extremely low frequency electromagnetic fields (ELF-EMF) and Alzheimer’s disease (AD), no reasonable mechanism can explain this association, and the related animal experiments are rare. In this study, ELF-EMF exposure (50Hz 400µT 60d) combined with D-galactose intraperitoneal (50mg/kg, q.d., 42d) and Aβ_25–35_ hippocampal (5μl/unilateral, bilateral, single-dose) injection was implemented to establish a complex rat model. Then the effects of ELF-EMF exposure on AD development was studied by using the Morris water maze, pathological analysis, and comparative proteomics. The results showed that ELF-EMF exposure delayed the weight gain of rats, and partially improved cognitive and clinicopathologic symptoms of AD rats. The differential proteomic analysis results suggest that synaptic transmission, oxidative stress, protein degradation, energy metabolism, Tau aggregation, and inflammation involved in the effects mentioned above. Therefore, our findings indicate that certain conditions of ELF-EMF exposure could delay the development of AD in rats.

## Introduction

Extremely low frequency electromagnetic field (ELF-EMF) is generated mostly by electric equipment, such as high voltage transmission lines, transformer substations, motors, and household appliances, with a frequency ranging from 0 to 300 Hz [1]. The most obvious problems with ELF-EMF biological effect studies are inconsistent and non-comparable results, which are possibly caused by different experimental parameters (frequency, flux density, and duration of ELF-EMF exposure) and subjects (cell lines, species, strains, sex, and age). Therefore, the dose-effect relationship and biological mechanisms of ELF-EMF are not fully understood.

Alzheimer's disease (AD) is an age-related progressive neurodegenerative disease characterized by progressive memory loss and a decline of cognitive function. The characteristic pathological changes of AD mainly include differing degrees of neuronal loss or apoptosis, senile plaques (SP) formed by extracellular deposits of amyloid-β (Aβ), and intracellular neurofibrillary tangles (NFT) constituted by hyperphosphorylated microtubule-associated protein tau (Tau) in the brain[2]. With the rapid growth of the elderly population in the world, the incident number of AD is also increasing year by year. AD represents a tremendous burden on patients, families, and societies.

Although some epidemiological investigations have shown a potential association between long-term exposure of ELF-EMF and AD[3–5], but few correlated animal experiments have been reported and no understood mechanism can reasonably explain this association. Over the past few years, various AD animal models have been established based on the possible mechanisms of AD. Previous studies have shown that D-galactose can cause premature aging and organ decline[6], and the intracerebral injection of Aβ_25–35_ peptide fragments can induce AD-like clinicopathologic features[7]. Therefore, ELF-EMF exposure combined with D-galactose intraperitoneal and Aβ_25–35_ hippocampal injection was implemented in this study to establish a complex rat model. (The injection treatment was base on the method used by Guo LL[8] and Liu S[9].) Through this model, we will have a better understanding of the relationship between ELF-EMF exposure and AD development, then to further explored its mechanism.

## Methods

### 2.1. Animal Elimination and Grouping

80 male Wistar rats aged 8 weeks (weight 212.6 ± 13.6g) were provided by the Experimental Animal Center of the Academy of Military Medical Science. According to individual difference in learning and memory ability, the Morris water maze training session(refer to section “**2.3. Morris water maze**”) was implemented to measure the average escape latency (AEL) of each rat before grouping. The outliers was figured out based on the Grubbs criterion[10] Then 16 corresponding rats were eliminated. The remaining 64 rats were randomly divided into control group (Con), magnetic field group (MF), Alzheimer’s disease group (AD), and complex model group (AD+MF). The animal use protocol was approved by the Animal and Human Use in Research Committee of the Academy of Military Medical Science. Rats were free with food and water, under the environment of constant temperature (23.0 ± 0.5°C) and humidity (65–75%) with a 12 h light/dark cycle (light on from 8:00–20:00).

### 2.2. Modeling

#### ELF-EMF exposure

**Fig 1:**
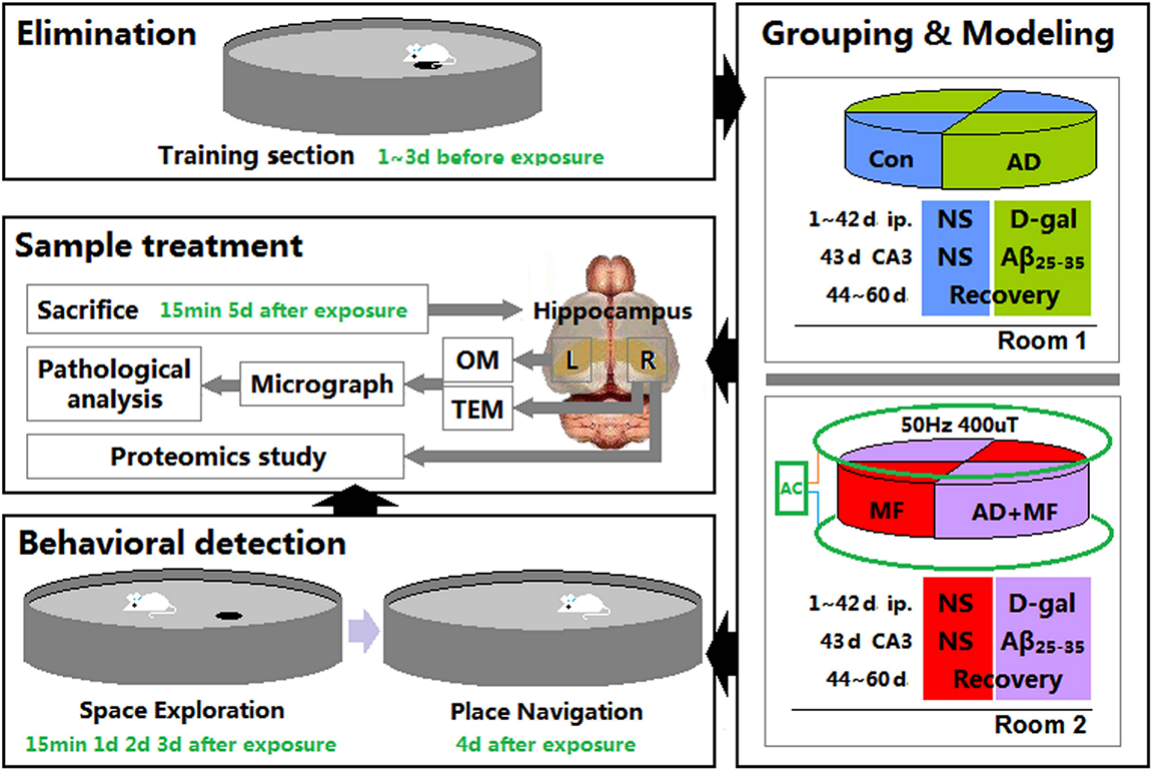
Flow Diagram of Experiment. The average escape latency (AEL) of 80 male Wistar rats were detected by Morris water maze (WMW) training session, then 16 outliers were eliminated. The remaining 64 rats were randomly divided into 4 group. 16 rats of each group were divided and 8 rats were kept in each box. ELF-EMF exposure system was installed in room2. When the exposure started, rats of AD and AD+MF groups received D-galactose intraperitoneal and hippocampal injection. Con and MF groups were injected with same volume of normal saline(NS). 15min and 5d after terminateion of 60d ELF-EMF exposure, 5 rats from each group were decapitated after becoming deeply anesthetized, and the hippocampus were collected. Then pathological analysis and proteomics study were performed. The learning and memory of 11 rasts from each group were detected by MWM test session.

The ELF-EMF animal exposure system was established by the Institute of Electrical Engineering, Chinese Academy of Sciences and consisted of voltage-regulator and two parallel coaxial circular coils with 70cm height, 140cm diameter. Two modular breeding devices were used in this research. Each one was comprised of 4 individual boxes with 20cm height, 50cm radius quadrant bottom. 16 rats of each group were divided and 8 rats were kept in each box (Fig 1). Group MF and AD+MF were placed in room2. which installed ELF-EMF exposure system. When the input current is 50Hz 1.65A, Gauss Meter(EFA300, Narda Safety Test Solutions GmbH, Germany) shows that the interior ELF-EMF intensity in breeding device is ringwise attenuating but longitudinal unchanged along the axis of the coils. It’s 50Hz and about 420uT at the vertex, 400uT at 25cm to the vertex, and 380uT at the edge of the box. Rats were continuously raised in the device, and exposed by ELF-EMF 24 hour a day for 60d. Group Con and AD were placed in room1, which was on the same floor and in the same ventilation system with room2. So the humidity, light, temperature, air quality of the two room were exactly equal. When the exposure system started in room1, the background magnetic field of room2 (involved, ELF-EMF created by electrical appliances, such as electric light, air conditioning etc.) was measured by Gauss Meter and identified below 400nT.

#### AD animal

**Fig 2:**
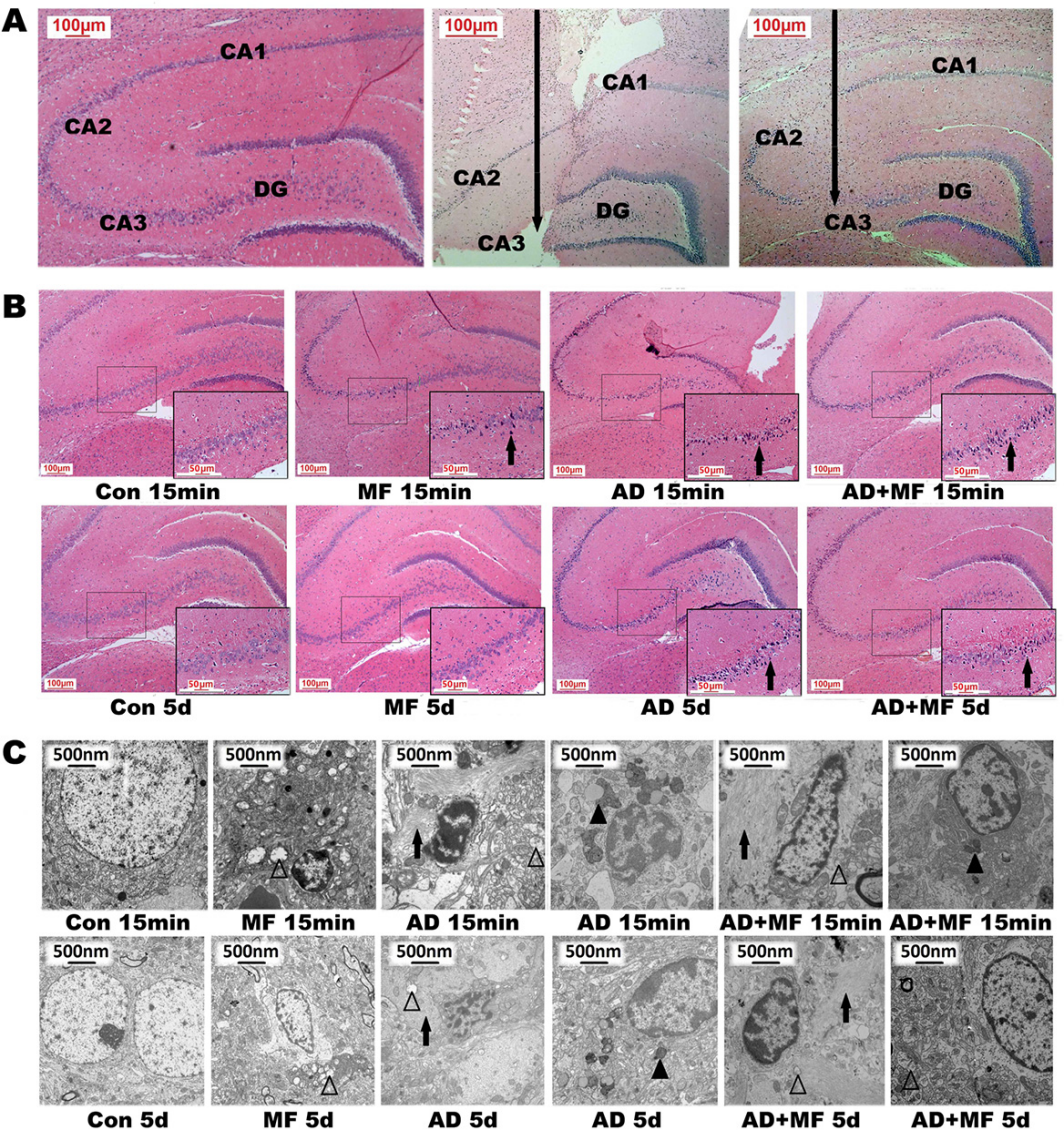
The hippocampal injection site and pathologic change in the CA3 region after exposure to 50 Hz and 400 μT of ELF-EMF for 60 days. (A-a): Hippocampal formation. (A-b, c): 1d and 7d after hippocampal injection, HE staining, →: injection site. (B) HE staining. CA3 region was enlarge in the right bottom box. →: nuclear contraction. (C) Electron microscope observation. →: neurofibrillary tangle; ▲:lipofuscin deposition; ∆: well and focal cavitations of mitochondria.

D-galactose (Sigma, USA) and Aβ_25–35_ (Sigma, USA) were dissolved with 0.9% NaCl saline to reach a concentration of 3 g/L and 1 μg/μl, respectively. Aβ_25–35_ was aggregated by in vitro incubation at 37°C for 7 days and then stored at 4°C. After the ELF-EMF esposure bigins, rats of AD and AD+MF groups received D-galactose (50 mg/kg) intraperitoneal injection once a day for 42 days. Con and MF groups were injected with the same volume of saline. At 43^th^ day, rats were anesthetized by intraperitoneal injection with 2% pentobarbital sodium (60 mg/kg), subsequently received hippocampal injection (5μl/unilateral, bilateral) of Aβ_25–35_ (AD and AD+MF group) or saline (Con and MF group) through a stereotaxic apparatus (BW-SDA903, Shanghai Bio-will Co. Ltd., China) and microinjection system (Ultra Micro Pump with SYS-Micro4 Controller, World Precision Instruments Inc., USA). Hippocampal injection was performed in the CA3 region(Fig 2) at the speed of 1 μl/min, and the orientation was AP, -3.5 mm; ML, ±3 mm; DV, 3.5 mm. Then rats were given an intramuscular injection of penicillin sodium (50,000 units) once a day for 3 days.

After the surgery, 3 rats of Con group, 1 of MF, 1 of AD and 2 of AD+MF died due to over anesthesia and postoperative infection. The other rats recovered well. The weight of each rat was recorded once a week during experiment.

### 2.3. Morris water maze

Morris water maze test is a behavioral procedure widely used in behavioral neuroscience to study spatial learning and memory[11]. In this study, the Morris water maze (SLY-WMS, Beijing Shuolinyuan Company, China) is a 180cm diameter circular swim pool that filled with 30cm depth water and be surrounded by lighttight curtain. Milk powder was added to muddy the water, and the temperature was kept at 25.0 ± 0.5°C. The water surface was divided into four quadrants, and an escape platform with 8cm diameter was placed at the center of quadrant I. The computerized tracking system and software (SLY-WMS, Beijing Shuolinyuan Company, China) was used to recorded the movements of rats. The MWM procedure refer to Vorhees CV, et al[11].

#### The MWM training session

This session was performed once a day for three consecutive days before ELF-EMF exposure. In this session, rat was put into water facing the wall in turn from quadrant I to IV. Rats were allowed to swim to search the platform for 60s. When boarded the platform, rats should be kept on it for 20s, then be put into next quadrant until finished the training of quadrant IV. Finally rats were dried with the towel and put back to the breeding device. The time that rats takes to find and board the platform was recorded as the escape latency (EL). If the rat can't find the platform in 60s, the EL was recorded as 60s. The average escape latency (AEL) of the four quadrants was calculated and used to animal elimination (refer to section “***2*.*1*. *Animal Elimination and Grouping***”).

#### The MWM test session

This session was performed once a day for five consecutive days after ELF-EMF exposure. The first four test sessions which called “place navigation trial” were similar to the training session mentioned above, except that the rats did not need to stay on the platform for 20s. Then the swimming speed and AEL of each rat was recorded. In the fifth test which called “space exploration trial”, the platform was removed, rats were put into the water from the edge of quadrant IV and allowed to swim for 60s, then the percent of time spent in quadrant I was recorded.

37 rats (8 in Con, 10 in MF, 10 in AD, 9 in AD+MF) participated this session. Then the outliers was eliminated based on the Grubbs criterion[10]to make the sample size in each group equivalent to 8.

### 2.4. Pathological analysis

15min and 5day after the termination of ELF-EMF exposure, 5 rats from each group were decapitated after becoming deeply anesthetized by 2% pentobarbital sodium (60 mg/kg i.p.). Then brains were collected and cut into left and right hemispheres.

4 left hemispheres from each group was fixed with 10% formalin and 5 μm paraffin sections were prepared. Then 2 Hematoxylin-Eosin (HE) and 2 Toluidine blue staining of each cerebral hemisphere was made and observed by microscope. The pathological image analysis system (CMIAS, Beijing University of Aeronautics and Astronautics, China) was used to quantitatively analyze the content of Nissl bodies in hippocampal CA3 neurons. The optical density of 2 visual fields, which were randomly selected from each Toluidine blue stained sections, were measured. Finally, the mean optical density (MOD) was calculated and used to judge the content change of Nissl bodies.

The others 3 unilateral hippocampus (1 left and 2 right from each group) was collected and cut into 1mm^3^ sections, fixed by glutaraldehyde and osmic acid, dehydrated by alcohol and acetone, embedded by resin, sliced into 40nm ultrathin sections, and stained by uranyl acetate and chromatic acid. The ultrastructural changes were observed under transmission electron microscope (TEM)(CM120, Philip, the Netherlands).

### 2.5. Proteomics study

15min after the termination of ELF-EMF exposure, 3 right hippocampus from each group was disrupted by ultrasonic disrupter (BL99-IIDL, Wuxi Voshin Instruments Co., LTD, China), then separately and sequentially processed through the following methods: Bradford method, immobilized pH gradients isoelectric focusing (IPGphor IEF System, Amersham Pharmaci Biotech Ltd, USA), SDS-PAGE (PROTEAN IIxi Cell, Bio-Rad, USA), silver staining, gel image scanning (ImageScanner, Amersham Pharmacia Biotech Ltd, USA), and images analysis (PDQuest 2-D Analysis Software 8.0, Bio-Rad, USA). Then the protein which was statistically different and has content ratio of >2 were considered to be differentially expressed. After in-gel protein digestion, the differential proteins were identified by peptide mass fingerprinting (PMF), peptide sequence tag (PST) technology, and a database search. Next, matrix-assisted laser desorption/ionization time-of-flight mass spectroscopy (MALDI-TOF-MS) measurements were performed on a Bruker Reflex III (Bruker Daltonik, Bremen, Germany), and nano ultra-high performance liquid chromatography electrospray ionization mass spectrometry tandem mass spectrometry (NanoUPLC-nano-ESI-MS/MS) was performed on a SYNAPT G2-S (Waters Inc., Manchester, UK). Then the database search was performed by the program MASCOT (http://www.matrixscience.co.uk), and peptide mass searches against the NCBInr database were performed for protein identification. Finally, the protein function was confirmed by a PubMed and UniProt database query.

### 2.6. Statistical analysis

The data were expressed as the mean ± standard deviation, and analyzed by the SPSS Statistics 17.0 software (SPSS Institute, USA). A value of *P <* 0.05 was considered to be statistically significant.

## Results

### 3.1. ELF-EMF exposure delayed the weight gain of rats

Weight data of 8 rats in each group were analyzed with three-factor (of which time was the repeated measurement) ANOVA analysis. Results (Fig 2) showed that ELF-EMF exposure significantly delayed the weight gain of rats (*P =* 0.001), but AD modeling has no significant effect on the weight (*P =* 0.898). The post hoc test suggest that compared with Con group, the weight gain of MF group significantly decreased (*P =* 0.017) fout weeks after the ELF-EMF exposure started. This change can be seen even one week early between group AD and AD+MF (*P =* 0.007).

### 3.2. ELF-EMF exposure improved the spatial learning disorder of AD rats

**Fig 3:**
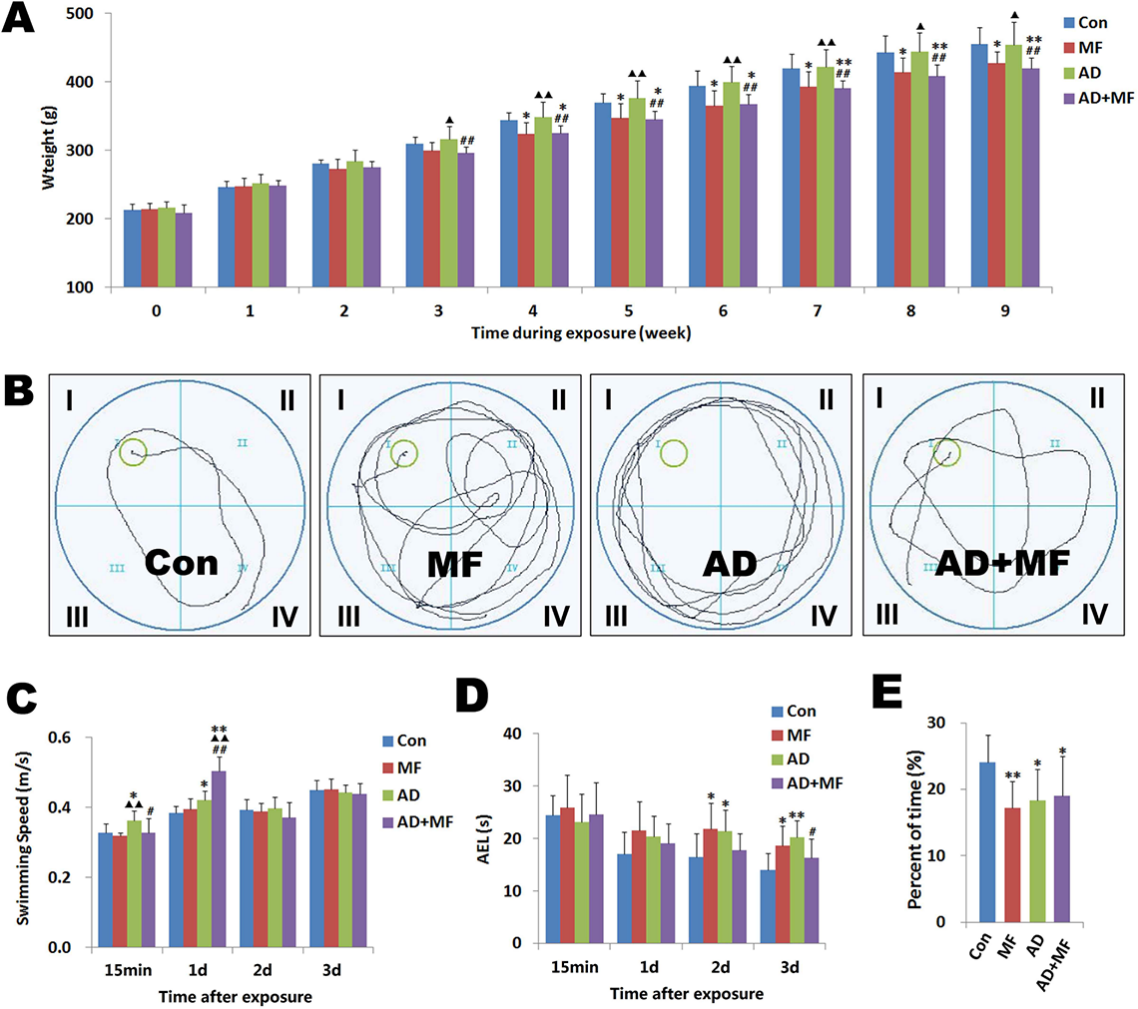
Weight and average escape latency (AEL) changes of rats during and after exposed to 50 Hz and 400 μT ELF-EMF for 60 days. (A) Weight changes of rats during exposure. Three weeks after ELF-EMF exposure started, compared with AD group the weight gain of AD+MF group significantly decreased (*P =* 0.007). This change can be seen at four week between group MF and Con (*P =* 0.017). (B) Swimming trajectory of rats 3 days after termination of exposure. The water surface was divided into I-IV quadrants, and the platform was positioned at the center of quadrant I. (C) Swimming speed changes of rats 0–3days after termination of exposure. (D) AEL changes of rats 0–3days after termination of exposure. (E) Percent of time spent in quadrant at 4day after termination of exposure. vs Con ** *P* < 0.01 * *P* < 0.05, vs MF ▲▲*P* < 0.01 ▲*P* < 0.05, vs AD *P* < 0.01 *P* < 0.05.

15min after terminated ELF-EMF exposure, AD rats were observed swimming quicker than the rats in group Con (*P =* 0.024), MF(*P =* 0.004) and AD+MF(*P =* 0.019). At 1d, AD(*P =* 0.020) and AD+MF(*P =* 0.000) group was significantly faster than Con group, and the AD+MF group even faster than AD(*P =* 0.000) (Fig 3). These results suggest that AD modeling may increased the anxious motion on rat.

At 2d and 3d, the AEL of MF (2d *P =* 0.018, 3d *P =* 0.013) and AD (2d *P =* 0.028, 3d *P =* 0.001) group were significantly prolonged than Con(Fig 3), and at 3d the AEL of group AD+MF was shorter than AD (*P =* 0.032). Three-factor (of which time was the repeated measurement) ANOVA analysis showed that ELF-EMF significantly improved the AEL extension of AD rats (*P =* 0.001). At 4d, the percent of time spent in quadrant I decreased significantly in the MF (*P =* 0.005), AD (*P =* 0.018), and AD+MF (*P =* 0.035) groups (Fig 3). Multi-factor ANOVA analysis further discovered that AD modeling obviously increased swimming speed of rats (*P =* 0.001). Otherwise, ELF-EMF exposure significantly relieved the extension of AEL (*P =* 0.001) and the contraction of time spent in quadrant I (*P =* 0.027) that induced by AD modeling.

It has been confirmed that repetitive training would help to form spatial memory. Rats were taught to remember the location of platform. Normally, AEL tend to decreased but the percent of time spent in quadrant I increased with the training times. So these two metrics have been considered as being prognostic with regard to spatial learning disorder[11]. Therefore these results suggested that ELF-EMF exposure might partially improved the spatial learning and memory disorder on AD rats.

### 3.3. ELF-EMF exposure improved the pathological damages of the hippocampus in AD rats

15min and 5day after ELF-EMF exposure, the HE staining sections of the four left hemispheres from each group were observed under microscope. Nerve injury changes such as nuclear contraction, cell edema, and perivascular space broadening was observed in CA1, CA3 and dentate gyrus(DG) region hippocampus within all treatment group (Fig 2).

Moreover, at 15min the typical pathological changes of AD included neuronal apoptosis, neurofibrillary tangles and lipofuscin deposition were observed in hippocampus of AD and AD+MF group through TEM (Fig 2). The other ultrastructure changes such as well and focal cavitations of mitochondria, dilation and degranulation of rough endoplasmic reticulum, and the fuzzy synaptic clefts, has been observed in all treatment groups. 5 days later, the injury mentioned above was about the same in group AD, but partly improved in group MF and AD+MF.

**Fig 4:**
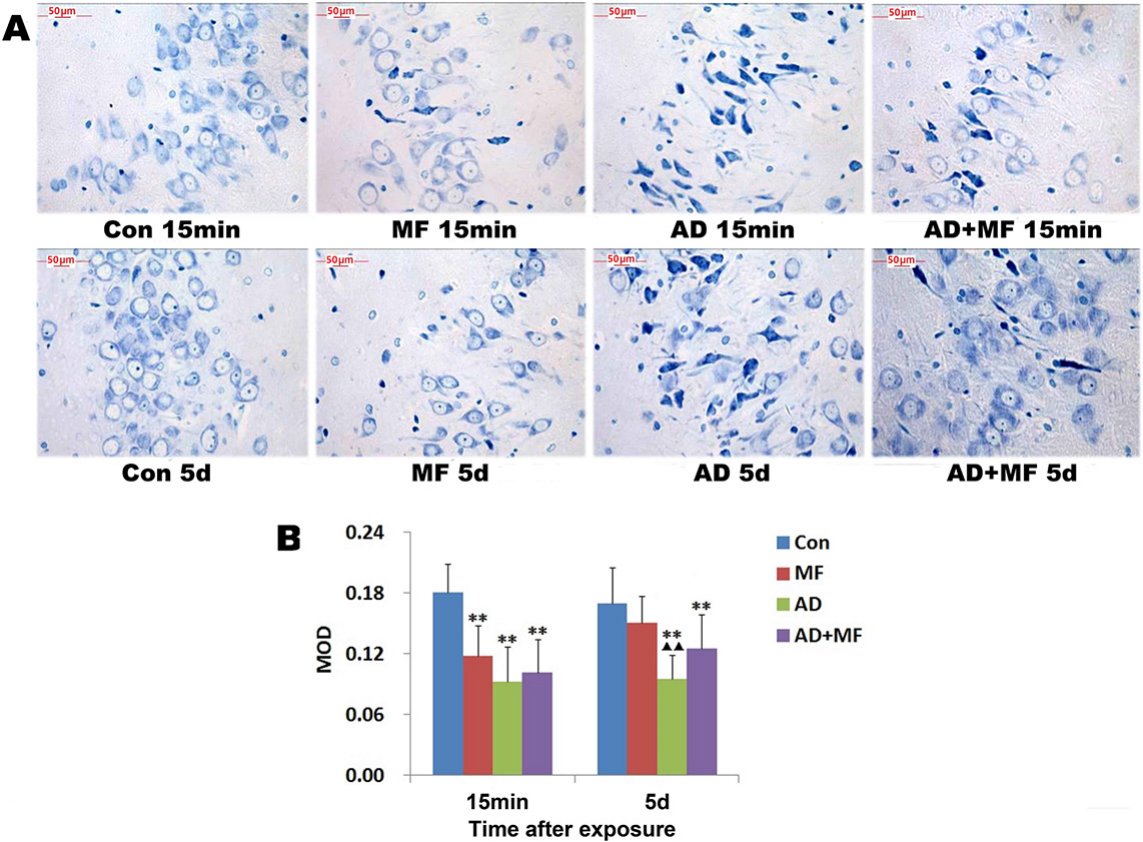
Nissl bodies changes in the CA3 region of rat hippocampus after exposure to 50 Hz and 400 μT of ELF-EMF for 60 days. (A) Toluidine blue staining. (B)Image analysis results of Nissl bodies. The mean optical density (MOD) was calculated and used to judge the content change of Nissl bodies. vs Con ** *P* < 0.01 * *P* < 0.05, vs MF ▲▲*P* < 0.01 ▲*P* < 0.05, vs AD *P* < 0.01 *P* < 0.05.

Quantitative analysis results shows that the Nissl bodies of hippocampal neurons significantly decreased in all treatment group (Fig 4) at 15min (*P* are all = 0.000) after the termination of ELF-EMF exposure. 5 days later, compared with group Con, the content of Nissl bodies was recovery in group MF, but still more lower in group AD (*P* = 0.000) and AD+MF (*P* = 0.006). The comparison of MOD at two time points show us further that Nissl bodies of MF group significantly increased at 5 day (*P* = 0.042). This result suggest the decrease of the Nissl bodies caused by ELF-EMF exposure was recoverable. Base on three-factor ANOVA analysis, we found that AD modeling can decrease the content of Nissl bodies (*P =* 0.042), and the ELF-EMF exposure can alleviate this decrease (*P =* 0.015).

### 3.4. Effects of ELF-EMF exposure on hippocampal protein profiling of AD rats

PDQuest 8.0 image analysis showed that six proteins were differentially expressed between the MF and Con groups instantly after ELF-EMF exposure, among which were four proteins that were up-regulated and two that were down-regulated in the MF group. There were four differential proteins between the AD and Con groups, one protein that was up-regulated and three that were down-regulated in the AD group. Eighteen proteins were differentially expressed between the AD+MF and AD groups; 11 proteins were up-regulated and seven were down-regulated in the AD+MF group (Table 1). These results suggest that ELF-EMF exposure resulted in more obvious changes of the hippocampal protein profile of AD model rats compared with normal rats, and that there were more up-regulated proteins. Furthermore, 15 proteins, which had both statistically difference and content ratio of >2 between any two groups, were identified by NanoUPLC-nano-ESI-MS/MS (9 proteins) or MALDI-TOF-MS (6 proteins) analysis (Table 2). The functions of the identified proteins are involved in synaptic transmission (SNCG, SNAP-25b), oxidative stress (CFL1, PRDX5, PRDX6), protein degradation (UCH-L1, UBE2N), energy metabolism (DUSP3, DDT, PDHE1-B, ECH), Tau aggregation (Tpi1, EFHD2), inflammation (FABP), and brain injury (MBP). These identified proteins may provide important clues for further investigation on the mechanisms of ELF-EMF exposure on AD development.

**Table 1:** The amount of differential proteins expressed in the rat hippocampus after exposure to 50 Hz and 400 μT ELF-EMF for 60 days.

Groups	Sta.	Ratio >2	Both	MF	AD	AD+MF
				↑ ↓	↑ ↓	↑ ↓
**Con vs. MF**	32	28	6	4 2		
**Con vs. AD**	37	62	4		1 3	
**AD vs. AD+MF**	18	63	18			11 7

**Table 2:** NCBI database results from 15 differential proteins identified by mass spectrometry.

Accession No.	Name	Score	Matches	Sequence coverage (%)	Theoretical pI	Mr (Da)
gi| 149035334	ubiquitin carboxy-terminal hydrolase L1, isoform CRA_b (UCH-L1)	104	11	47	5.05	24336
gi| 16758810*	ubiquitin-conjugating enzyme E2 N (UBE2N/Ubc13)	475	14(10)	32	6.13	17113
gi| 38512111	triose phosphate isomerase, partial (Tpi1)	155	11	46	7.07	26701
gi| 72255531*	EF-hand domain-containing protein D2 (EFHD2)	720	28(16)	48	5.01	26743
gi| 8393101*	cofilin-1 (CFL1)	86	3(2)	18	8.22	18521
gi| 16758404*	peroxiredoxin-5, mitochondrial precursor (PRDX5)	854	33(23)	38	8.94	22165
gi| 16758348	peroxiredoxin-6 (PRDX6)	128	11	64	5.64	24803
gi| 122066261*	Gamma-synuclein (SNCG)	116	4(1)	27	4.81	12969
gi| 1314856	synaptosomal associated protein of molecular mass25kD (SNAP-25b)	165	15	65	4.67	20572
gi| 149054346*	similar to Dual specificity protein phosphatase 3 (predicted), isoform CRA_b (DUSP3/VHR)	283	8(5)	24	5.79	23100
gi| 13162287*	D-dopachrome decarboxylase (DDT)	87	3(2)	23	6.09	13125
gi| 296439684	Pyruvate dehydrogenase E1 component subunit beta, mitochondrial (PDHE1-B)	115	14	47	4.63	22995
gi| 2392291*	Chain A, 2-Enoyl-Coa Hydratase, Data Collected At 100 K, Ph 6.5 (ECH)	396	11(8)	34	6.41	28269
gi| 13540630*	fatty acid-binding protein, brain (BFABP/ FABP7)	614	19(13)	56	5.46	14854
gi| 4454311	myelin basic protein (MBP)	109	11	69	11.75	14184

Proteins marked by “*” were identified by NanoUPLC-nano-ESI-MS/MS, and the remaining proteins were identified by MALDI-TOF-MS.

## Discussion

### 4.1. ELF-EMF parameters

Many researches have proven that the bio-effect of ELF-EMF is related to the magnetic frequency, flux density, and exposure duration. In China, the power source of appliances is 220 V and 50 Hz alternating current; therefore, people are most frequently exposed to 50 Hz ELF-EMF during daily living. Early reports have indicated that exposure to 100 μT and 50 Hz for 12 weeks had no effect on the learning and memory ability of rats[12]. Furthermore, 0.5 mT and 50 Hz of acute exposure treatments for 20 min showed no effect on the social and territorial behavior of rats, but may increase passivity and situational anxiety of the rats[13]. In addition, depressive-like behavior was found following 28–42 days of continued exposure[14]. A magnetic flux density that reaches 8 mT, 50 Hz, for 20 min may impair consolidation of spatial memory in rat[15], and 90 min[16] or 4 h[17] exposure showed devastating effects on the memory function of mice. According to the ICNIRP Guidelines (2010)[18], the occupational and public reference exposed threshold for 50 Hz ELF-EMF is 1 mT and 200 μT. In pre-experiment we found that 60d ELF-EMF exposure with 200uT 50Hz does not produce any significant effect in rats. So, we doubled the intensity, then the 400uT density was used in this study. In addition, 6 weeks consecutive peritoneal injection and 2 weeks postoperative recovery are needed to establish an AD rat model. Therefore, the parameters of the ELF-EMF exposure were 50 Hz, 400 μT, and 60 days in our study.

### 4.2. How should we define the bioeffect of ELF-EMF exposure—negative or positive?

In 1979, Wertheimer and Leeper[19] first reported the possible association between ELF-EMF exposure and childhood leukemia. Since that time, the negative effects of ELF-EMF was adopted and extensively researched. Epidemiological investigations identified that ELF-EMF exposure can cause the total decline of sleep quality[20], and some neurological symptoms, such as depression, anxiety, fatigue, and memory decline[21]. In addition, some reports have suggested the potential association between ELF-EMF and AD. For example, Schulte PA et al.[22] found that AD occurs more frequently in some occupations than in others. A study[23] further supported an increased risk of AD among employees occupationally exposed to ELF-EMF. Huss et al.[24] found that there was a dose-response relationship with respect to years of residence in the immediate vicinity of power lines and AD. In vitro studies confirmed that ELF-EMF exposure can influence the physiological function of nerve cells, such as the enhancement of oxidative stress[25], the receded function of neuronal ion channels and membrane receptors[26][27], the inhibition of proliferation, and the promotion of apoptosis[24]. ELF-EMF exposure can decrease the levels of neurotransmitters and related enzymes (serotonin, dopamine, gamma-aminobutyric acid, acetylcholine esterase)[28][29], and ultimately induce abnormal behavior and cognitive function disorders[14][17].

Meanwhile, various positive effects of magnetic field exposure have been reported. For example, ELF-EMF exposure (5 mT 60 min/days 12 days 50 Hz) can facilitate bone marrow stroma stem cell differentiation into neural cells[30]. ELF-EMF exposure (2 mT, 50 Hz, 1 h/days) from postnatal day 23–35 improved the spatial learning acquisition and memory retention of early adolescent male mice[31]. ELF-EMF exposure (80–150 μT, 40 Hz, 20 min/day, 5 days/week, 4 weeks) exerts positive effects on peripheral nerve regeneration[32]. ELF-EMF exposure (0.5 mT, 15 Hz, 12 h) significantly prolonged the window of opportunity for brain protection and enhanced the intensity of neuroprotection after traumatic brain injury[33]. ELF-EMF exposure (60 Hz, 0.7 mT, 21 days) improved neurological scores, enhanced neurotrophic factor levels, and reduced both oxidative damage and neuronal loss in a Huntington's disease rat model[34].

Experimental research on the association between ELF-EMF exposure and AD is rare. A single report suggested that continuous exposure of 100 μT and 50 Hz for 12 weeks had no effect on the pathogenesis of AD in aluminum-overloaded rats[12].

In previous experiments[35], we confirmed that recoverable damage of hippocampal morphology and spatial memory on rat can be induced by 400uT 50Hz 60d ELF-EMF exposure. But these damage can be partially recovered about a month after cessation of exposure. In this study, negative effect on weight gain, hippocampal neuron morphological structure, spatial learning and memory was obviously induced by both AD modeling and ELF-EMF exposure. However, the interaction between ELF-EMF and AD modeling should be received additional consideration. According to the results of multi-factor ANOVA analysis, the recovery function of ELF-EMF on extension AEL, contraction of time spent in quadrant I and content reduced of Nissl bodies on AD rats was identified.

All these suggest that ELF-EMF exposure may have opposite effects on health or sick animals. On the other hand, the reversibility of ELF-EMF damage may also contributed to their positive effects on AD rats.

### 4.3. The protein associated with the effect of ELF-EMF exposure on AD development

More than 100 years have passed since AD was first reported in 1906; the mechanism of AD is still unclear, and there is no effective therapy that can delay the onset or slow the progression of AD. It has been generally believed that the amyloid cascade and the abnormal posttranslational modification of Tau are the core of AD development[36]. In this study, the protein expression profile of the hippocampus in normal and AD rats were observed after ELF-EMF exposure. We found that the mechanism of ELF-EMF exposure on AD development relates to synaptic transmission, oxidative stress, protein degradation, energy metabolism, Tau aggregation, inflammation, etc. Moreover, many identified proteins have been reported to be involved in cognitive dysfunction, neurodegenerative disease, or brain injury.

For example, recent studies showed that UCH-L1 can alleviate the Aβ-induced synaptic dysfunction and memory loss in an AD model rat[37], and a lower expression of UCH-L1 was found in AD patients[38]. Furthermore, the co-localization of a novel amyloid protein, EFHD2, and pathological Tau in the brain of AD patients has been reported[39]. In addition, the interaction between Tau and TPI has been reported as follows: TPI can regulate glucose consumption, thereby inducing the intraneuronal aggregation of Tau[40], and phosphorylated Tau traps TPI and triggers the functional loss of TPI in the development of neurodegenerative diseases[41].

Oxidative stress is another familiar theory of AD development. Actually, the low expression of PRDX6 in the hippocampus, might contribute to the vulnerability of nitro-oxidative attacks in AD[42]. Under pathologic conditions, such as oxidative stress, an actin-binding and major actin depolymerization protein, CFL1, was found forming rod-like structures[43] that can mediate the loss of synapses, induce excess secretion of Aβ, and form neurofibrillary tangles, which might help to further tau modifications or assembly into paired helical filaments[44].

In addition, inflammation has been proposed to explain the plausible mechanisms of AD. Neuroinflammation is characterized by the activation of astrocytes and microglia as well as the release of proinflammatory cytokine and chemokine[45]. There have been reports that, in the brains of AD patients, the number and intensity of PRDX6+ astrocytes increased, especially around the neuritic plaques[46]. The abundant FABP+ astrocytes in the hippocampus have been frequently observed close to Ki67+ cells[47]; further studies have revealed that FABP can positively affect the proliferation of neural stem cells and negatively affect the survival of newborn neurons[48].

Otherwise, many more protein were also be thought to participate in the development of AD. For example, SNCG and SNAP-25b are both involved in regulating the structure and function of synapsis, and the mRNA expression level of SNCG increased[49] and SNAP-25b decreased[50] in AD patients. The expression of enzymes that are involved in cell metabolism, such as DUSP3 and PDHE1-B, changed in a dementia mouse model[51] and after Aβ_1–42_ treatment in vitro[52], respectively.

Altogether, some identified proteins in this study, such as UCH-L1, EFHD2, PRDX6, SNAP-25b, and DUSP3, may be important molecules for further investigating the mechanisms of MF exposure on the development of AD.

## Conclusion

In conclusion, our results showed that 50 Hz and 400 μT of ELF-EMF exposure for 60 days delayed the weight gain of AD rats, and partially improved the cognitive and clinicopathologic symptoms of the animals. Furthermore, several important molecular clues were provided in the current study. Further research is necessary to understand the molecular mechanism and fully assess the potential benefit of ELF-EMF exposure. This study indicated that ELF-EMF exposure could partially delay the development of AD in rats.
